# Care, management, and use of ferrets in biomedical research

**DOI:** 10.1186/s42826-024-00197-4

**Published:** 2024-03-26

**Authors:** Ravindran Kumar Pramod, Pravin Kumar Atul, Mamta Pandey, S. Anbazhagan, Suhas T. Mhaske, R. Barathidasan

**Affiliations:** ICMR-National Animal Resource Facility for Biomedical Research, Genome Valley, Hyderabad, Telangana 500101 India

**Keywords:** Ferret, Biomedical Research, Housing, Influenza, Cystic fibrosis, SARS-CoV-2, Toxicity

## Abstract

The ferret *(Mustela putorius furo*) is a small domesticated species of the family Mustelidae within the order Carnivora. The present article reviews and discusses the current state of knowledge about housing, care, breeding, and biomedical uses of ferrets. The management and breeding procedures of ferrets resemble those used for other carnivores. Understanding its behavior helps in the use of environmental enrichment and social housing, which promote behaviors typical of the species. Ferrets have been used in research since the beginning of the twentieth century. It is a suitable non-rodent model in biomedical research because of its hardy nature, social behavior, diet and other habits, small size, and thus the requirement of a relatively low amount of test compounds and early sexual maturity compared with dogs and non-human primates. Ferrets and humans have numerous similar anatomical, metabolic, and physiological characteristics, including the endocrine, respiratory, auditory, gastrointestinal, and immunological systems. It is one of the emerging animal models used in studies such as influenza and other infectious respiratory diseases, cystic fibrosis, lung cancer, cardiac research, gastrointestinal disorders, neuroscience, and toxicological studies. Ferrets are vulnerable to many human pathogenic organisms, like severe acute respiratory syndrome coronavirus 2 (SARS-CoV-2), because air transmission of this virus between them has been observed in the laboratory. Ferrets draw the attention of the medical community compared to rodents because they occupy a distinct niche in biomedical studies, although they possess a small representation in laboratory research.

## Background

Ferrets are small to medium-sized carnivorous mammals belonging to the family Mustelidae, which comprises weasels, martens, minks, stoats, badgers, and otters. Domestic ferrets (*Mustela putorius furo*) are most closely related to the European polecat (*Mustela putorius*) and deviated from the European polecat at least 2500 years ago.

**Table:** 

**Kingdom**: Animalia	**Phylum**: Chordata	**Class**: Mammalia
**Order**: Carnivora	**Family**: Mustelidae	**Subfamily**: Mustelinae
**Genus**: *Mustela*	**Species**: *M. putorius*	**Subspecies**: *M. p. furo* (Linnaeus, 1758)

The domestic ferret possesses short legs, long bodies, and thick fur with various body color patterns. In the laboratory, ‘fitch’ or ‘polecat’ ferrets appear with dark-colored guard hairs with a cream undercoat and a dark-colored mask, and points whereas albino ferrets show a single white colored coat. An adult ferret typically measures 51 cm in length and weighs between 0.7 and 2.0 kg. Male ferrets can be twice the size of females. It has a long tail with an average length of 13 cm. Because ferrets lack sweat glands and instead regulate their body temperature through panting plus additional behavioral processes, they are vulnerable to high temperatures and humidity. The other physiological parameters of the ferret are mentioned in Table 1.

**Table 1:** Physiological values of the domestic ferret

SI. No	General physiological parameters	Value
**1**	Weight	Male (hob): 1–2 kg Female (jill): 0.5–1.5 kg
**2**	Length	Approximately 51 cm, including the tail length of 13 cm
**3**	Longevity	5–10 years
**4**	Heart rate	200–350 beats/min
**5**	Respiration rate	33–36 times/min
**6**	Systolic blood pressure	Males: 161 mm Hg and Females: 131 mm Hg
**7**	Rectal temperature	38.8°C(37.8–40°C)
**8**	Daily water intake	75–100 ml
**9**	Food intake	5–7% of bodyweight
**10**	Sexual maturity	8–12 months
**11**	Fertility	Males: throughout lifespan and Females: 2–5 years (starting at 9–12 months)
**12**	Gestation period	41 days (39–42 days)
**13**	Reproductive cycle	Seasonally polyestrous with induced ovulation
**14**	Average litter size	8 kits (1–18)
**15**	Birth weight of the young one	6–12 g
**16**	Weaning age	6–8 weeks

Historically, ferrets were used for hunting mainly “ferreting” rodents, rabbits, and moles, as they are experts in getting down to the deepest holes and burrows. In the American West, these animals were widely used to protect grain from rodents between 1860 until the outbreak of World War II. As domestic pets, ferrets have gained popularity since the 1980s and 1990s. The cross between ferret and polecat, the ‘fitch ferret’ or polecat ferret, was used for fur production [1]. In 2008, a group of researchers developed genetically engineered ferrets [2]. Peng et al. (2014) reported the 2.41-Gb draft genome of the domestic ferret, which consists of 19,910 annotated protein-coding genes [3].

Various research approaches have been documented in ferrets, ranging from simple blood sampling to extensive invasive life-saving surgeries. Currently, the use of ferrets as biomedical research models is increasing day by day in respiratory diseases, toxicology, pharmacology, reproductive physiology, and endocrinology. Therefore, we aim to highlight the care, housing, breeding, and their uses in biomedical research.

## Main text

### Care, management, and breeding of ferrets

Ferret care and breeding procedures are similar to those of other carnivorous mammals maintained in laboratories in many aspects. It is an appropriate approach to house them in a group of two but not more than as it favors their social interaction and normal survival [4]. However, some ferrets may prefer to stay alone, and some species, such as the European polecat, prefer to remain solitary except in the first year between juveniles of the same litter and between males and females during the breeding season [5]. Considering this, special care should be taken to understand individual ferret preferences and make suitable housing arrangements accordingly. We will briefly describe an individual aspect of the care, management, breeding, anesthesia and euthanasia of ferrets herewith.

#### Housing

Because of their intense odor, ferrets should be housed in separate rooms from other species to avoid stress to those animals. The houses used for ferrets are known as hutches and are raised structures of rugged and durable ground materials, generally kept in an open shed. Each compartment in the wooden hutch should have a lid with a wire grid at the top to allow for proper air ventilation. Galvanized wire netting can be used for the floors to make cleaning easier. Loose solid boards should be placed on the wire grid floor for cleaning when soiled. A section of the wire flooring must be maintained clear of the nesting area so it can be used for urination and defecation. Feeding dishes should be made of either a galvanized or enameled rigid metallic sheet.

Several housing and conducive environment elements must be considered to establish a ferret as a suitable animal model in the laboratory. The differences lie primarily in the materials used to construct cages and diet formulation. Most ferret cages are made of a metal rod framework, much like those used to house rabbits. The flooring shall have durable metal cross bracing and a solid metallic platform to store feces and urine in a provided drop pan. If employing wire mesh or slatted flooring, the grid walls' separation should be 1.0 in. × 0.5 in. × 0.25 in. In social housing, rack interlinking creates more floor space and facilitates communication across cages. The welfare of the ferrets within the hutch depends on a better environment. They require access to secure hiding spots as well as objects that they can climb on, play with, and explore to maintain vigor and health, such as the use of metallic tunnels of polyvinyl chloride (PVC) piping to provide a playful time for ferrets in their daily routine [6]. Furthermore, the tunnel connected to two cages enhances social behavior.

The optimum recommended temperature range for ferrets in the laboratory is 15–21 °C (59–70°F) [7]. High humidity and extremely low temperatures must be avoided as they adversely affect their growth and reproduction. Also, underdeveloped sweat glands make it challenging to handle temperatures exceeding 30 °C (86°F). The optimum humidity should be between 40 and 65%. To maintain stock animals, the 12:12 light: dark cycle is preferable; however, breeding cycles can be controlled by manipulating the light hours. The estrus can be induced in females, and sexual activity can be signaled and attained in males throughout puberty via enhanced daylight or long-term exposure to artificial light in indoor-housed ferrets [8]. Ferrets kept in laboratory rooms should have ten to fifteen air exchanges of non-recirculated air per hour to control their musky odor [9]. The housing of ferret cages should be separate to avoid transmission of ferret odor to laboratory rodent species; otherwise, it causes fear among these animals, affecting their normal health status.

#### Feeding

A big mouth, characteristic carnivorous teeth, and short, strong jaws that make it easier to tear and chew food define the carnivorous digestive system of ferrets. Due to their strict carnivorous nature, ferrets require a meat-based diet rich in protein (30–40%) and fat and low in fiber and carbohydrates contents. Inadequate protein in the diet indeed leads to a negative nitrogen balance that causes poor growth and compromised reproductive potential. Feeding once or twice daily is better than offering ad libitum or feeding little. Ferrets shall be given small treats at frequent intervals as they are used to eating around ten small meals daily. Further, removing leftover food from the pen is necessary to avoid contamination and infection. Age, body weight, physical activity, reproduction, health, and the kind of food offered all affect the quantity of food needed. Supplying safe, fresh water on demand is essential. Water utensils should be cleaned regularly, and water should be served in stainless steel dishes or bottles with sipper tubes made of the same material.

#### Management

The ferrets are often handled without protective gloves by a trained handler. Wearing gloves is advised while performing physical examinations and administering medication to animals in a laboratory. The animal should be securely held at the nape with one hand while the other should hold the body. Hungry or ill ferrets can exhibit aggressive behavior and bite handlers. Ferrets become friendly when handled frequently, whereas early neutering reduces their likelihood of becoming aggressive. A ferret from a small group can be identified based on its body size, behavioral characteristics, or variation in coat color. Although fitch animals can be recognized by their appearance, seasonal variations in the pelage can make identification challenging. Identification in large groups is preferably done by individual markings or applying ear tags in animals, especially in groups [8]. Other identification methods are ear notching, ear tattooing, microchip implantation, and dye marking. Ferrets are highly susceptible to canine distemper (CD). Therefore, signs and symptoms of CD should be closely observed for a required period. All ferrets must be regularly vaccinated against CD. Animals must be vaccinated against CD, depending on the degree of biosecurity employed. Blood samples for hematology, serology, and biochemistry, as well as fecal screening for infections like Salmonella and Campylobacter, may be required for newly introduced ferrets whose health condition is unknown. Moreover, rabies vaccinations should be administered to the animals yearly from three months of age [10].

#### Breeding

Studies reported that successful breeding, gestation, and lactation of ferrets require a long photoperiod [11]. Ferrets reach puberty at approximately 8 to 12 months of age. In the winter, when the light hours are mostly less than 12 h, the physiological parameters in ferrets change because of less melatonin secretion. Less photoperiod exposure causes thick winter coat development with long hair and pale fluffy undercoats, gain in body weight, and hence, a sexual quiescence stage in both sexes. Light cycles play an essential part in the reproduction of the ferret and should be accounted for correctly when breeding is to be performed outside of the regular season [12]. A distinct increase in photoperiod is crucial, i.e., daily 8 h light increased to 16 h to maintain reproductive performance. Natural spectrum lighting, such as incandescent bulbs or fluorescent tubes, is capable of triggering alterations that promote sexual activity. Because the sensitivity to light varies among females, it is advisable to administer human chorionic gonadotropin (hCG) or gonadotropin-releasing hormone (GnRH) on the day the photoperiod is prolonged, to initiate breeding.

Both sexes respond roughly at the same time when high light intensity is employed to stimulate breeding, but the males might not be fertile enough to impregnate the females. Because of this, more care must be taken while breeding ferrets in artificial light or conducting research on them. Before extending the photoperiod for the group of females to be bred, males should be induced into breeding conditions. Males tend to have greasy skin secretions and a strong mustelid body odor during breeding. After completing a resting season of short photoperiod (6 weeks of 8 h of light per day), females can be brought into estrus by bringing them into a room on a 14-h light cycle. If young females raised on 6 to 8 h light cycles are switched to 14 to 16 h photoperiods, they will breed at 4–5 months. The ferret will become anestrous before reaching the complete breeding condition, so this transfer cannot occur before 90 days [13]. Mating occurs 10–14 days after the start of estrus, characterized by vulvar swelling [14]. The enlargement and complete descent of the testes into the scrotum indicate male breeding conditions.

The female in estrus should be taken to the male’s cage for breeding purposes, and the initial mating behavior should be observed to avoid any fighting or traumatic injuries. If hesitation is found, the male and female should be separated. Copulation is vigorous, noisy, and lasts from 10 min to 3 h. It is recommended to breed the female on two consecutive days to achieve a big litter size, even if spermatozoa remain in the female reproductive system for 36–48 h [15], as ovulation does not occur before at least 30 h. A progressive reduction in vulvar swelling over a few days indicates successful ovulation. The gestation period is 39 to 42 days. Typically, the domestic ferret gives birth to eight kits, each weighing between six and twelve grams. The kits should remain with their mother until they are at least eight weeks old, since they have a strong appetite. Hence, lactating mothers should be provided with a rich nutrient diet to nourish their young ones properly.

#### Anesthesia and analgesia

Understanding anesthetic drugs is crucial in managing ferret anesthesia, encompassing sedation, muscle relaxation, and analgesia. Administering anesthesia to ferrets follows protocols like those for dogs and cats. Fasting, recommended before anesthesia, should not exceed 8 h in young ferrets and 4 h in older ones due to their rapid gastrointestinal transit and the risk of hypoglycemia in middle-aged to geriatric ferrets. Gas anesthesia is considered the safest anesthesia method for critically ill ferrets, while injectable agents should be avoided in debilitated animals [16]. Gas anesthesia involves induction via 5% isoflurane or 7% sevoflurane using a face mask or induction chamber. Tracheal intubation, aided by a laryngoscope, is straightforward, and maintenance anesthesia often utilizes 3% isoflurane or 5% sevoflurane with an oxygen flow rate of 1 L/min, ensuring safe management of ferrets during procedures while considering their unique physiological traits [17]. Although many ferrets may exhibit hypersalivation during isoflurane induction, atropine is unnecessary as this behavior is typically transient [18].

For sedation and muscle relaxation, options like Acepromazine (0.1–0.3 mg/kg), Diazepam (0.5–2 mg/kg), Midazolam (0.5–2 mg/kg), Medetomidine (0.08–0.2 mg/kg), or Ketamine (5-15 mg/kg) are used, sometimes in combination. Anticholinergics like Glycopyrrolate (0.01 mg/kg) can support bradycardia. Analgesia can involve opioids like Buprenorphine (0.01–0.03 mg/kg), Butorphanol (0.1–0.5 mg/kg), Morphine (0.5 mg/kg), Hydromorphone (0.1–0.2 mg/kg), or non-steroidal anti-inflammatory agents such as Meloxicam (0.2 mg/kg SC or PO post-surgery). Lidocaine (< 2 mg/kg SC) and Tramadol (5 mg/kg PO q12h) are other options. Induction techniques may include Propofol (3–6 mg/kg IV), Ketamine (5–8 mg/kg IV) with Diazepam (1–2 mg/kg IV), Thiopental (8–12 mg/kg IV), or mask induction. Familiarizing oneself with the pharmacology of these drugs is essential before administering them to ensure safe and effective anesthesia management in ferrets [19–21].

#### Euthanasia

The selection of euthanasia techniques for animals is critical with the goal of minimizing pain and suffering while ensuring a compassionate and efficient procedure. In ferrets, various euthanasia methods are available based on established guidelines [22]. The recommended methods of euthanasia for ferrets encompass various approaches. The first and the most important method is asphyxiation using carbon dioxide (at a flow rate of 30%–70% of the chamber capacity per minute) followed by subsequent actions such as bilateral thoracotomy, decapitation, exsanguination, or major organ harvest [23]. The second method involves delivering an overdose of inhalant anesthetic such as isoflurane or sevoflurane until respiratory arrest occurs, which may be followed by bilateral thoracotomy, decapitation, exsanguination, or major organ removal. The third method is decapitation by guillotine, under sedation or anesthesia for juvenile or adult ferrets. This procedure without sedation or anesthesia in adult or juvenile animals requires a scientific justification. For newborns (less than seven days old), decapitation using sharp, well-maintained scissors or cervical dislocation is an approved physical means of euthanasia. The fourth technique is vital perfusion under injectable anesthesia, which includes inducing anesthesia with an injectable drug and then perfusing a specific solution into predefined vascular access points or blood egress sites. The next method of euthanasia is an injectable anesthetic (pentobarbital) overdose, which entails injecting a certain amount intraperitoneally and monitoring until heartbeats cease for a predetermined duration to ensure euthanasia. All these procedures should emphasize careful consideration of the ferrets' well-being, adherence to guidelines, and ethical protocols in the use.

### Ferrets in biomedical research

**Fig. 1:**
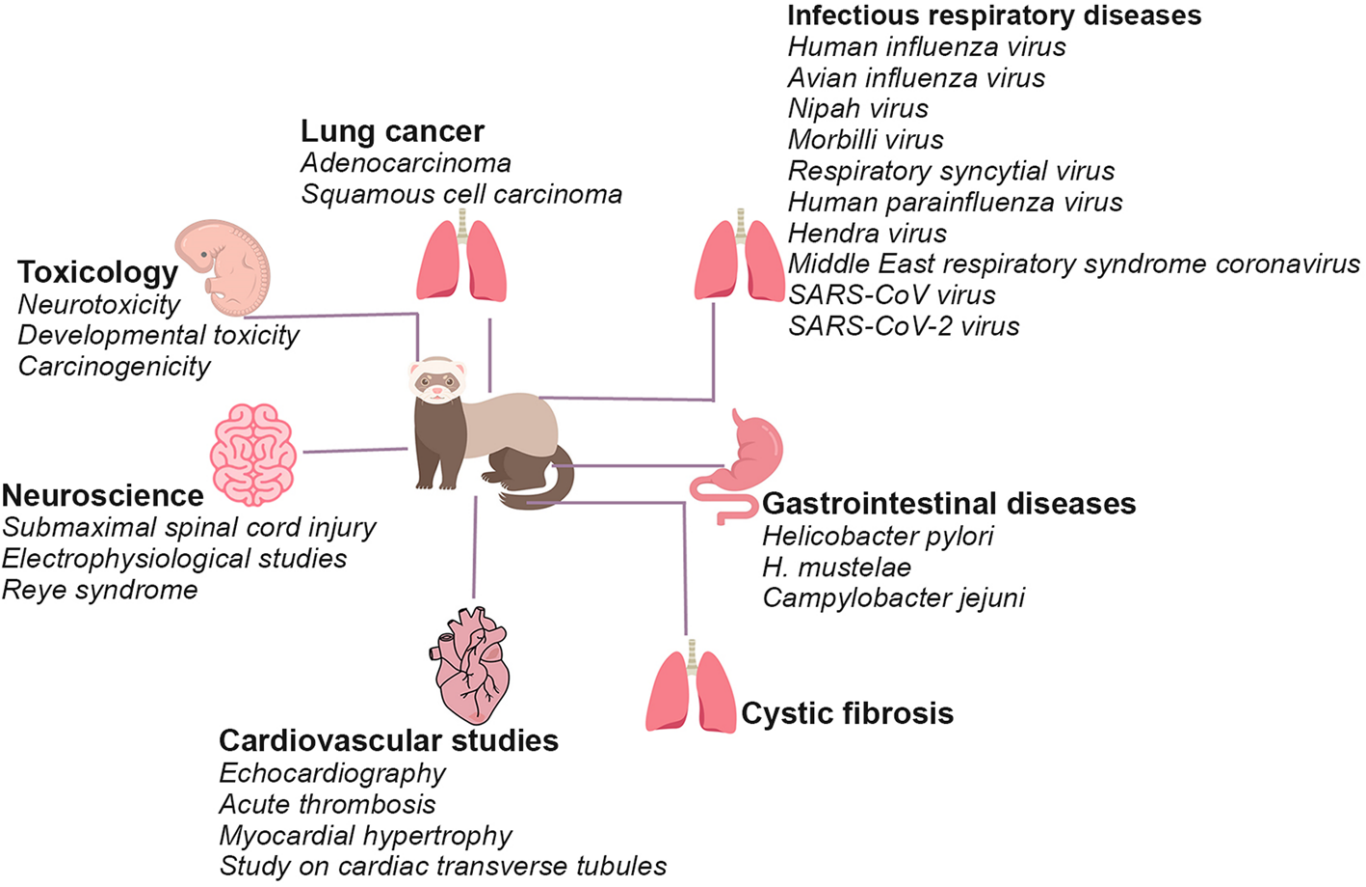
The use of ferrets in biomedical research. Ferrets were employed in studies on infectious respiratory diseases, gastrointestinal diseases, cardiovascular system, neuroscience, lung cancer, cystic fibrosis, and toxicity

In biomedical research, the use of ferret dates to the early 1900s, and the first study was published in 1911 [24]. Later, these animals were employed as study models for a number of conditions, including infectious respiratory diseases. Primarily, they have been used for lung cancer, cystic fibrosis, influenza, cardiac research, gastrointestinal diseases, neuroscience, and toxicological studies (Fig. 1). Their hardy nature, specific social behavior, diet and other habits, small size, and the requirement of a relatively low amount of test compounds and early sexual maturity (approximately eight months of age), in contrast to canines and non-human primates make them a suitable and substitute for dogs as well as rodents in biomedical research. Moreover, similarities in various anatomical, physiological, and metabolic features with humans, as well as their susceptibility to various human pathogens, are additional advantages in their usage [25]. Their ability to vomit is another essential characteristic that has made them the animal model of choice for evaluating prospective antiemetic drugs and identifying the emetic potential of future candidates in oncology. Additionally, ferrets are being employed in biomedical studies to investigate the sexual differentiation in the brain, physiological aspects of puberty and ovulation processes, and environmental variables influencing seasonal reproductive behaviors. Like humans, ferrets do not possess an inverted yolk sac placenta that is present in rodents and lagomorphs. Because of this characteristic, this species is a valuable animal model for teratogenicity research, especially for substances that act on the yolk sac and cause teratogenicity in rodents. Moreover, observations recorded on this species can be easily correlated to humans. Additionally, the ferret is susceptible to several known human teratogens, including methyl mercury, ethyl alcohol, thalidomide, vitamin A analogues, and alkylating anticancer agents. However, compared to rodent models, ferret models have a few drawbacks, such as more complicated husbandry requirements, higher costs, and minimal commercial availability (Table 2).

**Table 2:** Advantages and disadvantages of ferret model used for biomedical research

SI. No	Advantages	Disadvantages
1	Hardy nature	Complicated husbandry requirements compared to other animal models
2	Social behavior	Higher cost
3	Small size compared to non-rodent models	Minimal commercial availability
4	Relatively low amount of test compounds required compared to canine and non-human primate models	Enormous relative heterogeneity
5	Early sexual maturity (approximately eight months of age) compared to large animal models	Lack of availability of inbred and specific pathogen–free ferrets
6	Ability to vomit compared to rodents and lagomorphs: To study the emetic potential of future candidates in oncology	Shortage of contract laboratories with experience using them
7	Absence of inverted yolk sac placenta: Useful in teratogenicity research	Low number of quality breeders
8	Susceptible to several known human teratogens including methyl mercury, ethyl alcohol, thalidomide, vitamin A analogues, and alkylating anticancer agents	Lack of comprehensive databases
9	Upper and lower respiratory tracts similar to humans	
10	Ability to cough and sneeze	
11	Susceptible to unadapted human influenza virus isolates	
12	The ability to monitor viral shedding kinetics from both the upper and/or lower respiratory tracts using nasal washing or lower bronchoalveolar lavage	
13	Ferret *ACE2* (angiotensin-converting enzyme 2) gene is phylogenetically equivalent to that of humans	
14	Presence of outer subventricular zone (OSVZ) progenitor in brain similar to primates, especially in humans	

#### Infectious respiratory diseases

Ferrets are extensively used as research models in studying respiratory function and respiratory pathogens because of their similar upper and lower respiratory tracts to humans. In 1933, Smith et al. introduced ferrets as an animal model for influenza study [26]. After that, the ferret remains one of the most effective animal models for influenza studies because it is naturally susceptible to the human influenza virus with the development of similar symptoms that are seen in humans and can transmit the infection efficiently between individuals of the same animal group [27–29]. Other characteristics that facilitate and improve compatibility for respiratory research with this animal model are tracheal and lung anatomies, management of decreased airway resistance, relatively large lung capacity and their ability to cough and sneeze [30]. Human influenza virus types A and B naturally infect ferrets, offering an opportunity to explore the interplay of infection, illnesses, and amino acid sequence diversity in influenza virus glycoproteins in a perfectly controlled population [31]. Human and avian influenza viruses show identical binding patterns to sialic acids (the receptor for influenza viruses) in the respiratory tract [31, 32]. The specific expression of sialic acid, Neu5Ac, on the cell surface of ferrets, contributes to their usefulness as a model for the human-adapted influenza A virus [33]. However, sialylated glycan expression in ferret respiratory tract tissues differs from that in human tissues [34]. Because of the abovementioned features, ferrets are more susceptible to contracting certain influenza viruses and do not need to undergo an early host adaptation. As a result, ferrets can be employed for influenza research as well as the development of therapeutic and preventative candidates. The utility of ferrets in studies for the development of methods employing the measurement of viable influenza virus titer in droplets and droplet nuclei exhaled from infected animals has again established them as an excellent model [35]. Experimental inoculation of human and animal influenza virus by the intranasal route in ferrets resulted in elevated body temperature on the first day after inoculation, and the degree of pyrexia increased with the increase in viral titer, which continued for several days [36, 37]. Furthermore, Zitzow et al. demonstrated that, in contrast to the variable pathogenicity seen in BALB/c mice, H5N1 viruses are extremely virulent in the outbred ferret model [37]. Inagaki et al. reported that examination of nasopharyngeal swabs and nasal washes collected every 24–48 h from influenza virus-inoculated ferrets accurately represents virus replication in the upper respiratory tract [38]. Moreover, for a thorough investigation of several parameters commonly seen in severe human influenza infection cases, evaluation of white and red blood cell counts and serum chemical profiles in ferrets is recommended [38]. Research studies demonstrated that nasal wash or swab specimens from the ferret can be used to assess the inflammatory response in the upper respiratory tract, including the relative production of cytokines and chemokines [27].

In 2006, Maines et al. reported ferrets as effective comparative models for humans, as they parallel the effective transmission of H3N2 human viruses and the poor transmission of the avian virus, H5N1 [28]. Airborne transmission of the 2009 H1N1 virus between ferrets is determined by a single amino acid substitution of the virus [39]. Genetically altered H5N1 avian influenza virus strains that can easily transmit among ferrets are developed after multiple passages [39]. Moreover, the ferret has been extensively used with reassortant influenza viruses to identify the contributions of specific amino acids and gene segments to the virulence and transmissibility of the virus [40–42]. Proof of the role of T cells in heterosubtypic immunity came from ferret model studies. In these animals, heterosubtypic immunity was reported to develop against infections with H1N1 [43], H3N2 [44], H5N1 [45], or H2N2 [46], which was likely to be mediated by T cells. Recently, researchers have used ferret models to understand that treatment with the baloxavir drug in H1N1 infection reduced infectious virus release from the upper airways and the transmission frequency, even in delayed treatment conditions, i.e., two days after infection [47]. Again, this proves the usefulness of these species as a better model for studies on influenza viruses.

Besides their use to study human and avian influenza, they are also employed for research in numerous other respiratory infection-causing viruses, including Corona, Nipah, and Morbilli [48–50]. Researchers have reported the susceptibility of ferrets to severe acute respiratory syndrome-associated coronavirus (SARS-CoV) [48, 51]. When ferrets infected with SARS-CoV, they lost between 15 and 22% of their body weight [48, 51]. SARS-CoV-infected ferrets were affected with multifocal pulmonary lesions characterized by lymphocyte infiltration and alveolar damage without fever [51]. The weight loss is demonstrated as a reliable indicator of the general health of SARS-CoV-infected animals as the post-challenge weight gain of SARS-vaccinated animals was observed more than that of non-vaccinated animals. Using ferret as a model, Human monoclonal antibodies, as shown by ter Meulen and colleagues, may provide a practical and efficient prophylactic for managing human SARS coronavirus infection [52]. Chu et al. extensively optimized and characterized the ferret model used in their SARS-CoV experiments to validate clinical, histopathological, virological, and immunological endpoints [53].

Respiratory syncytial virus (RSV) is the most prevalent cause of newborn respiratory disease-related death in humans. Similar to humans, age-wise discrimination of RSV infection is also seen in ferrets, as adult ferrets show the presence of the virus only in the trachea and nasal turbinates after infection but not in the lungs [54], whereas infant ferrets show a rise in virus titer solely associated with alveolar walls [55]. Human parainfluenza virus type1 (HPIV1) and HPIV2 causing upper airway infection (croup) in children showed silent infections and developed specific neutralizing antibodies in adult ferrets [56]. In contrast, neonatal ferrets consistently succumbed to infection with HPIV3 that causes bronchiolitis and pneumonia in humans. Hutchinson et al. demonstrated that ferrets are an excellent model for studying Zika virus infection in brain tissue [57]. With differing degrees of success, numerous types of other viruses, such as the human parainfluenza viruses [58], Hendra virus [59], Nipah virus [60], and Middle East respiratory syndrome coronavirus [61], have also been investigated in the ferret model.

#### Contribution of ferrets to severe acute respiratory syndrome coronavirus 2 (SARS-CoV-2) studies

**Fig. 2:**
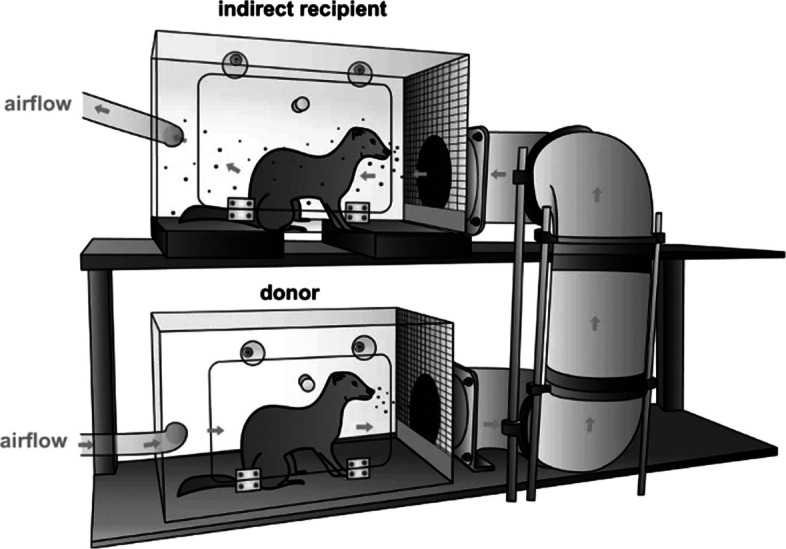
Schematic representation of the setup used to assess transmission of SARS-COV-2 virus among ferrets [65]

Ferrets have recently been used for the experimental inoculation of SARS-CoV-2. The ferret *ACE2* (angiotensin-converting enzyme 2) gene is phylogenetically equivalent to that of humans, and their ACE2 protein makes six hydrogen bonds with the SARS-CoV-2 S protein, resulting in a significant binding force [62]. Many researchers have reported the efficient air transmission of SARS-CoV-2 between ferrets [63–65]. The same pattern of virus shedding was noted by Richard et al. in the direct contact/indirect recipient with the inoculated ferrets. Because of this, using the ferret as a model helps us better understand the molecular underpinnings of SARS-CoV-2 and other betacoronavirus transmissibility, as well as the mechanism of transmission [64]. According to reports, ferrets may be mildly or asymptomatically infected with SARS-CoV-2 [65, 66]. The virus can replicate in the upper airway tract of ferrets for up to eight days, mainly in the tonsils, soft palate, and nasal turbinates [67]. The therapeutic effect of MK-4482/EIDD-2801 (ribonucleoside analogue inhibitor of influenza viruses) to control SARS-CoV-2 has been demonstrated using a ferret model [68]. Kutter et al. demonstrated that airborne transmission of both SARS-CoV and SARS-CoV-2 over more than a meter distance is possible in ferrets [65] (Fig. 2). Only mild clinical disease was presented for SARS-CoV-2 infection in ferret models; thus, Ryan et al. employed a ferret model for performing dose titration studies of SARS-CoV-2 [69]. According to a recent study using a ferret model, delivering peptides that match the highly conserved heptad repeat domain at the C terminus of the S protein (HRC peptides) intravenously two days before cohousing with an infected animal for 24 h completely protected the animals from infection and demonstrated efficacy against mutant viruses [70].

#### Lung cancer

The pulmonary structure and airways of ferrets are similar to those of humans, making them ideal for studying numerous aspects of lung disorders, including lung cancer. Kim et al. developed lung tumor in ferrets by exposing them to both 4-(methylnitrosamino)-1-(3-pyridyl)-1-butanone (NNK) and cigarette smoke [71]. In another investigation, the same team demonstrated that β-carotene in the presence of α-tocopherol and ascorbic acid protected both preneoplastic and neoplastic lesions, resulting from 6 months of tobacco smoke exposure and NNK treatment [72]. In addition, NNK exposure alone resulted in preneoplastic lesions (squamous metaplasia, dysplasia, and atypical adenomatous hyperplasia) and tumors (squamous cell carcinoma, adenocarcinoma, and adenosquamous carcinoma) in ferrets, which are prevalent in humans [73]. Because ferrets imitate human lung cancer, they are a promising model for studying lung carcinogenesis and exploring potential interventions and treatments. Furthermore, they offer numerous opportunities for identifying biomarkers and investigating molecular mechanisms in lung cancer research.

#### Gastrointestinal diseases

The Ferret gastrointestinal system is anatomically and physiologically similar to humans and harbors *Helicobacter pylori*, which causes most duodenal and gastric ulcers in humans. Many researchers have successfully used the ferret as a model for studying the *H. pylori*-like organism *H. mustelae* [74–76]. Even though the colonization pattern of the ferret model differed from that of *H. pylori* in humans, it has been widely utilized in vaccination investigations. In 1987, Fox et al. used the ferret as an animal model for human campylobacteriosis [77]. A few years later, through the experimental infection study in ferrets, Bell and Manning reported immunity against *Campylobacter jejuni*-induced enteric disease [78]. This study demonstrated some humoral immunity that protects against enteric disease development but without colonization of *C. jejuni*. Anorexia, dehydration, and bacteremia with greenish mucoid stools and occult blood were observed 24 h after the challenge for C. *jejuni* in the ferret model [79]. In addition, the virulence capability of mutants of the pspA gene (associated with the expression of a *C. jejuni* pilus-like appendage) was also assessed in this species [79, 80].

Taking advantage of having an emetic reflex compared with rodents and lagomorphs [81], numerous studies on vagus and gastric physiology have been conducted on ferrets [82–84]. Using a ferret model, Nanivadekar and colleagues recently developed a methodology to identify gastrointestinal disorders and symptoms like nausea and vomiting using machine learning algorithms to recognize the physiological state of the stomach and the beginning of retching [85].

#### Cystic fibrosis

In 2010, Sun et al. reported a cystic fibrosis (CF) ferret model for the first time, and they reported a 75% incidence of meconium ileus at birth [86]. The CF kits had a 50% perforation rate due to the meconium ileus location, which was in the distal part of the small and large intestine. On the other hand, CF ferrets do not have a macroscopically identifiable ileocecal junction marker. Meconium dilatation and distal microcolon in ferrets were shown to be comparable to those in newborn humans. Distal intestinal obstruction syndrome (DIOS)/constipation was reportedly treated prophylactically in CF transmembrane conductance regulator-knockout ferrets, but the medical intervention was used to manage the occasional DIOS/constipation episodes [87]. The variety of fecal bacteria across pairs of ferrets with and without cystic fibrosis (CF) appears to be genotype-independent, supporting the findings of Sun and colleagues that the environment plays a significant influence on the gut microbiome [87]. Ferrets with CF were distinguished from non-CF ferrets by distinct bacterial species; also, CF ferrets had greater levels of aerobic and anaerobic bacterial titers from the small intestine, which may be due to compromised immunity. According to research, neonatal ferrets exhibit early alterations in liver enzymes without histological proof of cholestasis, like CF infants [88, 89].

Sun et al. noticed mild pancreatic disease in CF ferrets at birth, characterized by multifocal acinar lumen dilation and lesser ducts by eosinophilic zymogen material [86]. One research group reported the suitability of ferrets for investigating CF-related diabetes because they showed pancreatic destruction, which is associated with glycemic instability and progressive glucose intolerance [90]. VX-770 (Ivacaftor) is an FDA-approved drug for clinical application to patients with CF. Recently, Sun et al. developed ferrets with a VX-770 (ivacaftor)––responsive CFTRG551D mutation to study the impact of CF in utero pathologies. They demonstrated that the pancreas, intestine, and male reproductive tract were partially protected from developmental pathologies by the administration of VX-770. Homozygous CFTRG551D/G551D animals showed the most effective VX-770-mediated protection against these pathologies [91].

#### Cardiovascular studies

The small size, discrete nodal masses, and unique coronary arterial pattern made the ferret heart an ideal model for histochemical, ultrastructural, electrophysiological, pathological, and blood circulation studies, as several groups used ferrets as models for cardiovascular studies [92]. The comparison of a clinically normal ferret with a ferret with an experimentally induced right ventricular hypertrophied heart was conducted to study the heart rate, presence and magnitude of S waves, and total voltage (sums of positive and negative deflections in lead I, II, and III) [93]. Normal values of the cardiac silhouette, M-mode, and Doppler echocardiogram [94, 95] and conscious stage ECG and echocardiography [96] of the ferret heart have already been established. Acute thrombosis and myocardial hypertrophy have also been studied using ferret heart [97, 98] and demonstrated differentially regulated pre-translational, translational, and post-translational levels of Na( +)-K( +)-ATPase isoforms in the hypertrophied heart [99]. Caldwell et al. used ferrets along with sheep and rat models to study the reliance of cardiac transverse tubules on the BAR domain protein amphiphysin II (AmpII) and reported that AmpII is intricately associated with t-tubule maintenance [100].

#### Neuroscience

In 1976, Eidelberg et al. reported the ferret as a model of submaximal spinal cord injury by direct thoracic cord compression [101]. Furthermore, researchers compared the antiepileptic drugs (E)-2-[(amino)phenylmethylen]-benzo [b] thiophen-3(2H)-on (AF-CX 921 XX) and carbamazepine (CBZ) using lissencephalic ferret models [102]. The relationship between patterns of electrical activity and behavior has been extensively studied in the ferret model by performing electrophysiological experiments [103, 104]. Because of a gyrencephalic cortex, ferrets also possess outer subventricular zone (OSVZ) progenitors present in primates, especially in humans [105]. The relative developmental immaturity of the neonatal ferret also makes it easier to study how early lesions in one area of the brain affect connectivity in other areas, as well as how lesions affect the formation of topographical maps and connectivity between the cerebral hemispheres [106, 107]. In 2016, Sukhinin et al. developed a database of anatomical connections and architectonic features of the ferret brain, i.e., the Ferret(connect)ome (www.Ferretome.org) [108]. This database further assisted the use of ferrets in electrophysiological or developmental studies. Reye syndrome is a rare and potentially fatal pediatric illness recognized as acute noninflammatory encephalopathy with fatty liver failure. The ferret is additionally employed as an animal model for investigating Reye syndrome. Reye’s syndrome is a severe condition that affects children between the ages of 6 and 12 and is induced by the consumption of aspirin and other anti-inflammatory drugs, primarily affecting the brain and liver. In ferrets, symptoms of the disease appear only after administering a diet imbalanced in arginine, followed by infection with human influenza type B virus and therapy with aspirin. Similar to children, affected ferrets experience encephalopathy symptoms that progress and ultimately result in coma and death [109, 110].

#### Toxicology

Ferrets are one of the non-rodent animal models used in toxicology and drug development since the 1970s. Brantom et al. used ferrets as a model for studying the toxicity of Orange G (1-Phenylazo-2-naphthol-6,8-disulfonic acid disodium salt) in 1977 [111]. In 1985, this animal model was used to assess the pharmacokinetics of ethanol [112]. In addition, many researchers have used ferrets for developmental toxicological studies [113–116]. European ferrets were proposed as a possible mammalian model for studying organophosphorus delayed neurotoxicity, as Tanaka et al. (1991) reported delayed neurotoxicity of bis (1-methylethyl) phosphorofluoridate (DFP) on the central nervous system of this species [117]. Using observations reported during toxicity trials on ferrets, researchers assessed the effects of alcohol on the fetus during the third trimester of human gestation [118]. The ferret was also found significant in the study of the effect of developmental alcohol and valproic acid exposure on the play behavior of the ferret [119]. These studies displayed the ferret as a model of greater importance for investigating the fundamental mechanisms of social interaction. Researchers used ferret to study the carcinogenic effect of 4-(N-methyl-N-nitrosamino)-1-(3-pyridyl)-1-butanone (NNK), a tobacco carcinogen that causes lung tumors in this species upon injection [73]. Similar to humans, ferrets also displayed preneoplastic lesions (squamous metaplasia, dysplasia and atypical adenomatous hyperplasia) and tumors (squamous cell carcinoma, adenocarcinoma and adeno squamous carcinoma) on NNK exposure.

## Conclusions

Based on the aforementioned facts from the cited articles, the ferret is considered an important laboratory animal in biomedical research and particularly useful for studies on viral infections. Furthermore, ferrets are phylogenetically more similar to humans than mice or rats, therefore this animal is a more valuable model system than non-human primates as they present new opportunities to deepen our understanding of human biology. Moreover, it is the only animal model that exhibits clinical symptoms after influenza infection that are equivalent to those of humans. Recently, researchers have reported the efficient use of ferrets in SARS-CoV-2 studies. They are good non-rodent models for biomedical research because of their hardy nature, social behavior, diet, small size thus the requirement of comparatively fewer test substances, and early sexual maturity compared with dogs and non-human primates. Due to many practical reasons, such as comparatively low cost, sociability to allow group housing, body dimensions and minimum space requirement, they are suitable substitutes for canine model test systems in cardiac studies, neurological studies, pharmacological trials and toxicity testing.

## Data Availability

All data associated with the review were mentioned in the Manuscript.

## Abbreviations

CD: Canine distemper
CF: Cystic fibrosis
DIOS: Distal intestinal obstruction syndrome
FDA: Food and Drug Administration
GnRH: Gonadotropin-releasing hormone
g: Gram
h: Hour
hCG: Human chorionic gonadotropin
HPIV: Human parainfluenza virus
IV: Intravenous
kg: Kilogram
mg: Milligram
min: Minute
ml: milliliter
NNK: 4-(N-methyl-N-nitrosamino)-1-(3-pyridyl)-1-butanone
OSVZ: Outer subventricular zone
PO: Per os
PVC: Polyvinyl chloride
RSV: Respiratory syncytial virus
SARS-CoV: Severe acute respiratory syndrome-associated coronavirus
SARS-CoV-2: Severe acute respiratory syndrome coronavirus 2
SC: Subcutaneous
